# Dietary factors and risk of SARS-CoV-2 infection in the Moli-sani Study Cohort: Marialaura Bonaccio

**DOI:** 10.1093/eurpub/ckac130.235

**Published:** 2022-10-25

**Authors:** M Bonaccio, A Di Castelnuovo, S Costanzo, M Persichillo, A De Curtis, F Gianfagna, MB Donati, G de Gaetano, C Cerletti, L Iacoviello

**Affiliations:** 1 Department of Epidemiology and Prevention, IRCCS NEUROMED, Pozzilli, Italy; 2 Cardiocentro, Clinica Mediterranea, Naples, Italy; 3 Department of Medicine and Surgery, University of Insubria, Varese, Italy

## Abstract

**Background:**

A healthy diet plays a major role in supporting the immune system which is critical to protect the host from pathogenic organisms. To date, evidence on the relationship between dietary habits and the risk of SARS-CoV-2 infection is still scarce.

**Methods:**

Analyses on 1,096 participants from the Moli-sani Study (2005-2010) who were re-examined in 2017-2020, and in January-September 2021. Food intake was assessed in 2017-2020 using a 188-item FFQ. Adherence to Mediterranean diet (MD) was evaluated using the Mediterranean Diet Score (MDS) ranging from 0 to 9. Multivariable logistic regression models were used to estimate odds ratios (OR) and 95% confidence intervals (95%CI) for incident SARS-CoV-2 infection in association with dietary factors.

**Results:**

Out of 1,096 participants, 90 either reported to have tested positive for COVID-19 or were positive for anti-SARS-CoV-2 antibodies before receiving any COVID-19 vaccine. In a multivariable-adjusted model controlled for known risk factors, a 1-point increase in MDS was associated, though not significantly, with lower risk of SARS-CoV-2 infection (OR = 0.90; 95%CI 0.78-1.04). Among individual dietary components, a high consumption of vegetables or fruits and nuts was associated with lower odds of SARS-CoV-2 infection (OR = 0.57; 0.34-0.96 and OR = 0.61; 0.37-1.00, respectively). High fish intake was otherwise linked to increased risk of infection (OR = 2.05; 1.25-3.36). Nutritional factors associated with reduced risk of infection were dietary fibre (OR = 0.50; 0.27-0.93 for 10 g/d increase), vegetable proteins (OR = 0.56; 0.33-0.94 for 10 g/d increase) and vitamin C (OR = 0.94; 0.89-0.99 for 10 g/d increase).

**Conclusions:**

Adherence to MD was suggestive of a lower risk of SARS-CoV-2 infection. In particular, large amounts of fruit and vegetables were associated with reduced odds of being infected, as well as diets rich in fibre, vegetable proteins and Vitamin C.

**Key messages:**

- Diets rich in vegetables, fruits, Vitamin C and fibre were independently associated with lower risk of SARS-CoV-2 infection.
- Nutrition could represent an effective strategy at the population level to contribute to the protection against SARS-CoV-2 infection.

